# Impacts of the Urbanization Process on Water Quality of Brazilian Savanna Rivers: The Case of Preto River in Formosa, Goiás State, Brazil

**DOI:** 10.3390/ijerph120910671

**Published:** 2015-08-31

**Authors:** Nayara Luiz Pires, Daphne Heloisa de Freitas Muniz, Tiago Borges Kisaka, Nathan de Castro Soares Simplicio, Lilian Bortoluzzi, Jorge Enoch Furquim Werneck Lima, Eduardo Cyrino Oliveira-Filho

**Affiliations:** 1 Programa de Pós-Graduação em Meio Ambiental e Desenvolvimento Rural (PPG-MADER), da Faculdade UnB Planaltina (FUP), Universidade de Brasília, CEP, 73300000, Planaltina, DF, Brazil; E-Mails: nayara.pires@ifg.edu.br (N.L.P.); daphne.muniz@embrapa.br (D.H.F.M.); tiagobk.df@gmail.com (T.B.K.); nathan.simplicio@gmail.com (N.C.S.S.); 2 Department of Education, Universidade Estadual de Goiás—UEG, CEP 73807250, Formosa, GO, Brazil; E-Mail: libortoluzzi@yahoo.com.br; 3 Laboratório de Ecotoxicologia, Embrapa Cerrados, CEP 73310-970, Planaltina, DF, Brazil; E-Mail: jorge.werneck-lima@embrapa.br; 4 Centro Universitário de Brasília—UniCEUB, CEP 70790075, Brasília, DF, Brazil

**Keywords:** urban sewerage, water sources, water contamination, macrophytes

## Abstract

The release of domestic sewage in water resources is a practical feature of the urbanization process, and this action causes changes that may impair the environmental balance and the water quality for several uses. The aim of this study was to evaluate the influence of urbanization on the surface water quality of the Preto River throughout the town of Formosa, Goiás, Brazil. Samples were collected at five points along the river, spatially distributed from one side to the other of the town of Formosa, from May to October of 2012. Data were subjected to descriptive statistics, as well as variance and cluster analysis. Point P2, the first point after the city, showed the worst water quality indicators, mainly with respect to the total and fecal coliform parameters, as well as nitrate concentrations. These results may be related to the fact that this point is located on the outskirts of the town, an area under urbanization and with problems of sanitation, including absence of sewage collection and treatment. The data observed in this monitoring present a public health concern because the water body is used for bathing, mainly in parts of Feia Lagoon. The excess of nutrients is a strong indicator of water eutrophication and should alert decision-makers to the need for preservation policies.

## 1. Introduction

Although 70% of the surface of Earth is covered by water, only 3% of it is fresh water, distributed in aquifers, polar ice caps, rivers, lakes and other reserves such as clouds and steam; 97% is saltwater [1]. On a worldwide scale, Brazil is lucky to have the greatest reserve of fresh water on the planet [2]. However, despite its wealth in rivers, there are problems of contaminated water in various cities, resulting in a range of negative consequences for biodiversity in general and for the quality of human life.

Water resources in the Brazilian Cerrado comprise an important region of springs between the great Brazilian hydrographic regions. Near the springs, rivers generally have lower carrying capacity and dilution of effluents, which amplifies the importance of knowledge about the major risks that occur in areas undergoing urbanization to guide monitoring plans, investments in sanitation infrastructure and integrated management of land use and water resources. The agricultural development of the past 40 years has stimulated the rapid growth of cities and the urbanization process until the present day. In general, the sanitation sector cannot match the speed of the urbanization process, generating environmental liabilities [3,4]. 

**Figure 1:**
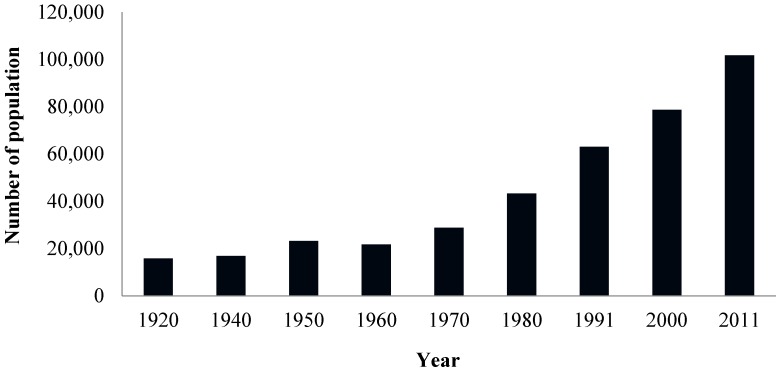
Population growth in the county of Formosa from 1920–2011 [6,7].

Some of the sources of the Preto River basin are around the town of Formosa, in the state of Goiás. A few kilometers downstream from Formosa, after Feia Lagoon, the Preto River represents the natural boundary between Goiás state and the Federal District, where its hydric resources are used intensively for irrigation purposes. Before its confluence with the Paracatu River, the main tributary on the left bank of the São Francisco River, the Preto River is still used to generate energy by means of the Queimados Hydroelectric Plant, which is in the state of Minas Gerais. Therefore, a loss in water quality may affect not only part of the population of Formosa, but also impact the irrigation channels in the Federal District that are closest to the town, due to the influence of the urbanization process which is happening so quickly in this region. In Formosa City the population is high and has grown exponentially over the last 40 years (Figure 1).

Contamination of water by urban centers mainly occurs via outflow of domestic sewerage systems, which is not only constant and highly polluting, but is also related to the spread of human water-borne diseases [5]. At this point, the investigation of the river water quality is very important for public health policies, mainly because Josefa Gomes Creek and Feia Lagoon are widely used by the population of Formosa City for bathing. 

Then, the aim of this study was to evaluate the influence of urbanization on the characterization of surface water of the Preto River throughout the town of Formosa, Goiás, Brazil, from its headwaters (source) to Feia Lagoon. This is a pioneering study in this region, and it involves an unprecedented analysis of the environmental situation of an important river located in the Midwestern region of Brazil. 

## 2. Materials and Methods 

### 2.1. Study Area 

**Figure 2:**
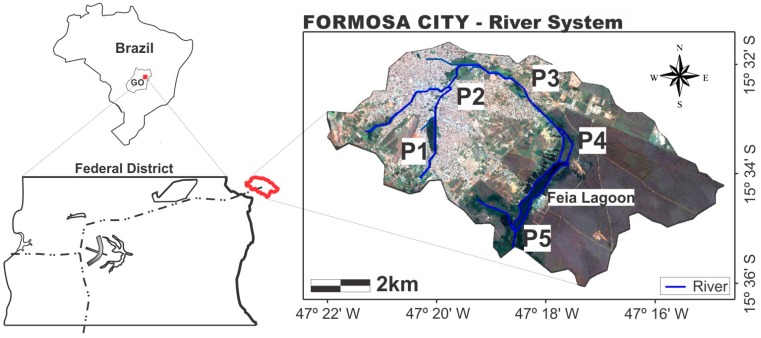
Map showing the location of the headwaters of the Preto River in Formosa, Goiás, with respective collection points. **Source:** satellite image (Landsat-8) modified by SPRING 5.0 software.

Throughout the hydrographic basin that forms the headwaters of the Preto River, in Formosa City, samples were collected at five geo-referenced points, called, respectively, P1, P2, P3, P4 and P5 (Figure 2). 

### 2.2. Analytical Methodologies

The parameters temperature, DO, pH, TDS and EC were determined in the field, in unfiltered samples, using a multi-parameter portable HI 9828 measure (Hanna Inst., Woonsocket, Rhode Island, USA). Turbidity tests were carried out in the laboratory, on the collection day, with a turbidimeter model HI 93703 (Hanna Inst., Woonsocket, Rhode Island, USA). Analysis of total hardness was done in the laboratory using the titulometric method by EDTA-Na [8]. In samples filtered through a membrane filter (0.45 μm), anion content was determined for fluoride (F^−^), chloride (Cl^−^), nitrite (NO^2−^), nitrate (NO^3−^), bromide (Br^−^) phosphate (PO_4_^3−^) and sulfate (SO_4_^2−^); and for cations: lithium (Li^+^), sodium (Na^+^), ammonium (NH4^+^), potassium (K^+^), calcium (Ca^2+^) and magnesium (Mg^2+^), by ion chromatography using the *761Compact* IC chromatograph (Metrohm AG, Switzerland). To guarantee quality assurance and quality control of measurements, calibration standards were prepared each day of analysis, using Merck® reagents. Blanks, duplicates, and spiked samples were used too. Calibration coefficients were maintained at least to three 9’s before proceeding with samples (r = 0.999). The analyses of total and thermotolerant coliforms were performed using the chromogenic method (Colilert®, Idexx, Westbrook, Maine, USA). The methodologies used in the laboratory follow the proposal in Standard Methods for the Examination of Water and Wastewater [9]. 

### 2.3. Statistical Analysis

The quantitative data were obtained by analyzing the variables of water, pH, conductivity, TDS, DO, hardness of water, turbidity, total coliforms, *Escherichia coli,* Na^+^, K^+^, Ca^2+^, Mg^2+^, F^−^, Cl^−^, NO_2_^−^, NO_3_^−^ and SO_4_^2−^ . Statistical analyses were performed using the software R.

Data were initially submitted to descriptive statistics. Analysis of the parameters in the collected samples took into consideration the totality of the periods of the data, *i.e*., the months of May, June, August and October, using as measurements the mean and the standard deviation, as well as the maximum and minimum values attributed to the variables.

After describing the data, the Kolmogorov-Smirnov normality test was applied at a significance level of 5% by means of the “ks.test” function [10,11]. Furthermore, aiming to analyze the variability of the data in function of the sampled points and the seasonality represented in the study, an Analysis of Variance (ANOVA) was carried out with the variables distributed on the normality curve.

ANOVA tests the hypothesis H0, which is the possibility of all the means of the analyzed treatment being equal. For this, the F test is used as presented in the Analysis of Variance table. When the F test value is higher than the tabled F, the null hypothesis is rejected, thus showing significant differences between at least one pair of means in the treatment [12]. In this study, the function “aov” [13] was used with a 5% degree of freedom.

In addition, Cluster Analysis was applied, with the purpose of viewing the standard distribution of the sampling points in function of the variables [14]. For this, the variables that presented parametric distribution were standardized by the method “standardize”. In this case, the variables have their value subtracted from the mean and divided by the standard deviation, and then present zero mean and standard deviation equal to one [15]. With the standardized variables, a distance matrix of the Euclidean similarity index was created. Definition of the clusters was carried out using the UPGMA algorithmic procedure, by means of the “hclust” function.

## 3. Results and Discussion 

### 3.1. Physical-Chemical and Biological Analyses

Among the data from physical and chemical analyses, the results of analysis of temperature, EC, TDS, turbidity, pH, DO and water hardness at the sample points are summarized in Table 1.

**Table 1:** Result of the physical and chemical parameters analyzed in the surface water in the headwaters of the Preto River, Formosa, GO, Brazil.

Collection Points	Temp. (*) (°C)	EC (µS·cm^−1^)	TDS (mg·L^−1^)	Turbidity (NTU)	pH	DO (mg·L^−1^)	Hardness (mg·L^−1^ CaCO_3_)
**Collection date: *May 07***							
**P1**	23.5	44	22	0.11	5.9	2.3	9
**P2**	24.7	137	68	0.78	7.2	5.6	34
**P3**	21.9	128	64	1.18	7.4	6.3	45
**P4**	(**)	(**)	(**)	(**)	(**)	(**)	(**)
**P5**	24.1	154	77	0.02	6.9	3.3	61
**Collection date: *June 25***							
**P1**	23.2	35	18	2.29	5.8	3.2	9
**P2**	24.1	138	69	5.29	7.4	5.6	36
**P3**	20.2	124	62	1.86	7.7	5.3	41
**P4**	19.5	128	64	4.94	7.7	5.1	44
**P5**	18.7	161	81	1.43	6.7	3.4	69
**Collection date: *August 13***							
**P1**	19.7	128	64	2.52	6.3	3.5	12
**P2**	23.5	163	82	3.98	7.2	5.1	37
**P3**	18.8	131	65	2.12	7.2	5.3	41
**P4**	18.1	135	68	3.08	7.6	4.7	46
**P5**	22.1	161	81	3.72	6.4	4.4	73
**Collection date: *October 01***							
**P1**	(***)	(***)	(***)	(***)	(***)	(***)	(***)
**P2**	27.4	165	83	1.95	7.5	6.7	45
**P3**	23.6	163	81	5.12	7.8	7.4	56
**P4**	22.8	168	84	1.88	7.7	4.9	59
**P5**	25.9	159	79	3.24	7.6	3.1	81

Notes: (*) Temp. = temperature; (**) the analyses were not carried out due to errors in the localization of the point (P4) in the field; (***) the analyses were not carried out due to the small volume of water at this point.

After carrying out chromatographic analyses, it was not possible to detect the ions Li^+^, Br^-^ and PO_4_^3−^ in significant quantities in the collected samples. The ion NH_4_^+^ was detected only in P2, in August 2012, with a concentration of 0.36 mg·L^−1^. The results of the concentration of the other ions are presented in Table 2.

**Table 2:** Result of the concentrations of ions analyzed in the surface water at the headwaters of the Preto River, Formosa, GO.

Points	F^−^ (mg·L^−1^)	Cl^−^ (mg·L^−1^)	NO_2_^−^ (mg·L^−1^)	NO_3_^−^ (mg·L^−1^)	SO_4_^2−^ (mg·L^−1^)	Na^+^ (mg·L^−1^)	K^+^ (mg·L^−1^)	Ca^+^ (mg·L^−1^)	Mg^+^ (mg·L^−1^)
**Collection date: *May 07***									
**P1**	0.04	2.43	n.d.	2.28	0.30	7.32	0.34	4.01	0.40
**P2**	0.05	7.03	0.06	13.85	0.90	15.03	1.95	8.50	1.22
**P3**	0.05	4.06	n.d.	0.17	0.48	11.76	0.57	8.46	1.38
**P4**	(*)	(*)	(*)	(*)	(*)	(*)	(*)	(*)	(*)
**P5**	0.03	1.58	n.d.	n.d.	0.45	4.01	2.14	12.28	4.63
**Collection date: *June 25***									
**P1**	0.05	2.75	n.d.	2.53	0.57	7.26	0.41	4.59	0.45
**P2**	0.09	7.59	0.10	13.81	1.13	14.38	2.19	8.51	1.40
**P3**	0.07	4.95	n.d.	0.16	0.67	11.53	0.51	9.05	1.42
**P4**	0.02	4.99	n.d.	0.15	0.74	11.84	0.72	9.79	1.55
**P5**	0.01	1.27	n.d.	n.d.	0.35	3.11	1.99	10.37	5.65
**Collection date: *August 13***									
**P1**	0.06	5.05	n.d.	0.77	0.66	10.36	0.28	3.50	0.33
**P2**	0.11	8.72	0.09	13.69	3.80	16.21	2.33	10.43	1.40
**P3**	0.03	5.09	n.d.	0.19	0.56	11.99	0.55	13.26	1.35
**P4**	0.08	5.49	n.d.	n.d.	0.63	12.17	0.64	14.53	1.61
**P5**	0.09	1.19	n.d.	n.d.	0.40	2.34	2.22	15.98	6.28
**Collection date: *October 01***									
**P1**	(**)	(**)	(**)	(**)	(**)	(**)	(**)	(**)	(**)
**P2**	0.11	8.62	0.24	12.17	2.10	16.06	2.20	14.05	1.77
**P3**	0.04	5.88	n.d.	0.15	0.34	13.01	1.10	15.52	1.86
**P4**	0.05	5.98	n.d.	0.08	0.46	12.92	1.43	15.71	1.98
**P5**	0.02	0.65	n.d.	n.d	0.07	1.89	2.11	12.79	7.10

Notes: (*) the analyses were not carried out due to errors in the localization of the point (P4) in the field; (**) the analyses were not carried out due to the small volume of water at this point; (n.d. = not detected).

**Figure 3:**
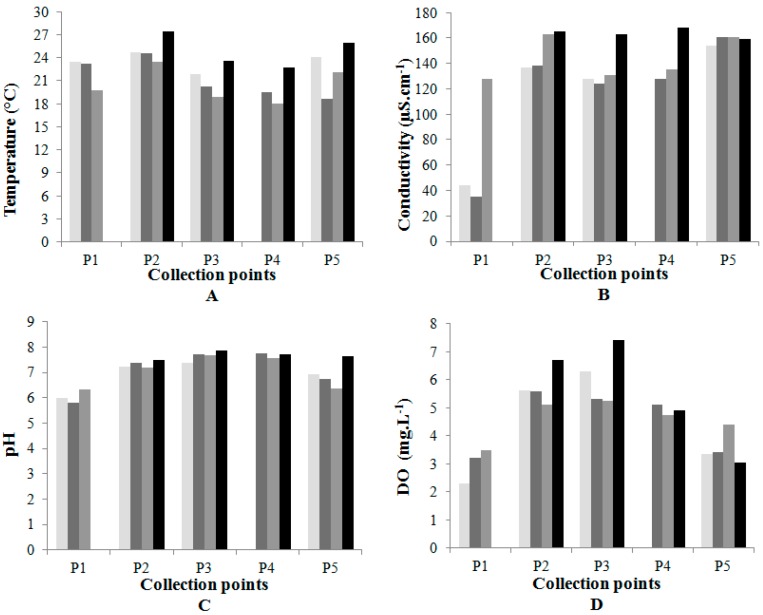
(**A**) Temperature; (**B**) Electrical conductivity; (**C**) hydrogen ion potential and (**D**) dissolved oxygen.

Water temperature, represented in Figure 3A, presented the highest values at point P2 during the sample collections. An increase in surface water temperature is generally provoked by sewage outflows [13].

The EC values shown in Figure 3B were higher than 100 µS·cm^−1^ at points P2 to P5. An increase in EC is observed in a water body that receives untreated domestic effluents, and levels of EC above 100 µS·cm^−1^ suggest negative environmental impacts [16].

A reduction in the pH value in a water body functions as an indicator of imbalance in the ecosystem. The values found in P5, Figure 3C, may be an indication that the ecosystem of this region is environmentally unbalanced [16].

Points P2, P3 and P4 presented a greater quantity of DO, with values almost always over 5 mg·L^−1^, as can be seen in Figure 3D. These values are due to the incorporation of O2 along the watercourse. The low value of DO at P1 is due to the short contact time with the atmospheric air and with photosynthesizing organisms. At P5 it is due to an environment with little current and probably due to the consumption of oxygen by heterotrophic organisms, because the higher the quantity of organic matter introduced into the water, the greater the quantity of oxygen consumed by it, besides being a lentic environment.

**Figure 4:**
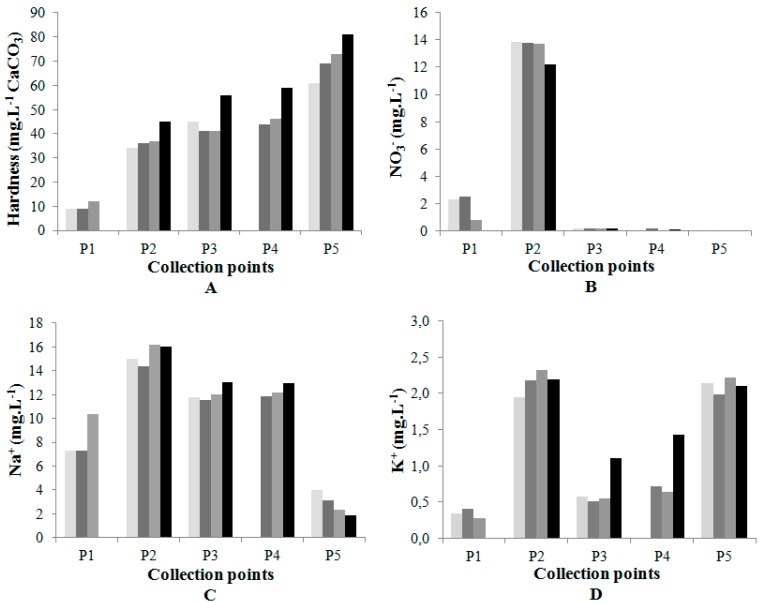
(**A**) Total hardness; (**B**) nitrate (**C**) sodium and (**D**) potassium.

Water hardness is mainly related to the content of Ca^2+^ and Mg^2+^, a fact that was observed in this work, since the increase in the level of these elements was accompanied by an increase in hardness, shown in Figure 4A [9]. The low hardness at P1 (source) is probably related to the low content of calcium and magnesium in the soils of the Cerrado region, as also observed by other studies [5,17]. The concentrations of magnesium in Cerrado natural surface waters are generally around or lower than 1 mg/L [3]. At P5 all the samples showed concentrations above of these baseline values. 

Nitrate, as shown in Figure 4B, was what presented the highest concentrations among the nitrogenated compounds studied. At P2 significant values were detected, indicating contamination by sewage flowing into the Beira Rio Creek, also responsible for the increase in sodium concentrations, shown in Figure 4C at this point, as well as observed in other studies [5]. The presence of nitrate in P1, the spring of the river, in May and June, can be due to the runoff of this compound derived from the litter decomposition [18,19,20].

Potassium, shown in Figure 4D, is essential for the nutrition of plants, and in the form of ions is rapidly assimilated by them. In Josefa Gomes Creek a large number of aquatic macrophytes were found, and these are likely to consume the potassium in this stretch of the river, explaining the low concentrations of potassium at points P3 and P4.

Toothpaste, medicines, vitamins and chewing gum are also sources of F^−^ [9]. The high concentrations found mainly at P2, P4 and P5 may be associated with contamination from domestic sewage, increasing the naturally occurring concentrations of this chemical element.

Table 3 presents the Most Probable Number (MPN) of total and thermotolerant coliforms obtained in 100 mL of water samples. It can be observed that the points P2, P3 and P4 presented a high index of total coliforms during all analyses (>2419.6 MPN/100 mL). At P5 it was only in the last collection (October 2012) that the value was lower than this, totaling 1732.9 MPN/100 mL of total coliforms.

**Table 3:** Results of the biological parameters in the surface water at the headwaters of the Preto River, Formosa, GO.

Date	P1	P2	P3	P4	P5
**Total coliforms (NMP/100 mL)**					
07/05/2012	1732.9	>2419.6	>2419.6	(*)	>2419.6
25/06/2012	1299.7	>2419.6	>2419.6	>2419.6	>2419.6
13/08/2012	920.8	>2419.6	>2419.6	>2419.6	>2419.6
01/10/2012	(**)	>2419.6	>2419.6	>2419.6	1732.9
**Thermotolerant coliforms (NMP/100 mL)**					
07/05/2012	307.6	>2419.6	248.1	(*)	33.1
25/06/2012	98.4	>2419.6	648.8	727.0	18.7
13/08/2012	261.3	1986.3	365.4	770.1	290.9
01/10/2012	(**)	>2419.6	613.1	166.4	107.1

Notes: (*) The analyses were not carried out due to errors in the localization of the point (P4) in the field; (**) the analyses were not carried out due to the small volume of water at this point.

The high density of thermotolerant coliforms in the P2 water samples indicates a high level of contamination by sewage, given that these microrganisms come from the intestine of humans and other warm-blooded animals, and are eliminated in large numbers with feces (106/g–108/g) [9]. The presence of these bacteria indicates that this water is unsuitable for human consumption [21].

At P5 large quantities of the macrophyte water hyacinth (*Eichhornia crassipes*) were noted. Water hyacinth is recognized in the literature as an indicator of polluted environments, and its presence is associated with eutrophy since it is the species that develops best in such environments [22].

Another aquatic plant species found in large quantities along Josefa Gomes Creek was Southern Cattail (*Typha domingensis*), which is typically present along the banks of drainage channels, lakes and reservoirs, and on várzea floodplains [23]. In general, the growth of macrophytes is associated with an increase in the availability of nutrients, especially nitrogen and phosphorous, and with a greater amount of light due to the removal of gallery forest [4].

**Figure 5:**
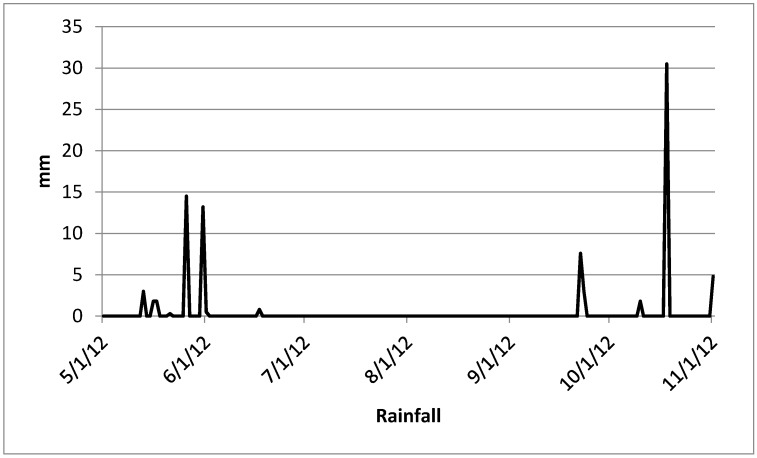
Rainfall in the studied period monitored by the meteorological stations from Embrapa Cerrados. Source: Laboratory of Environmental Biophysics at Embrapa Cerrados.

To relate the obtained results with the season, Figure 5 shows the rainfall values in the studied area, based on the monitoring of the meteorological stations from Embrapa Cerrados.

Figure 5 shows that the collection days (7 May, 25 Jun, 13 Aug and 1 Oct) there were not rainfall in the study area and then is possible to concluded that the results were not influenced by the rain presence, but the dry season may have been responsible for the reduction of some values such as nitrate and potassium in P1, suggesting that the observed quantitative may actually be coming from runoff.

In the studied area it can be noted that superficial drainage occasioned by the topography of the city and the constructed micro-drainage encourage the flow of rubbish into the water body. Most of the county of Formosa has no rainwater drainage system. The capture and movement of rainwater directs the flow to Josefa Gomes Creek, altering the water quality, carrying a large amount of solid residues and provoking instability on the banks of the source, compromising aquatic life. Such problems related to the urban drainage system affect various Brazilian cities and occur in function of environmental, political, social and economic aspects [24]. These characteristics are present in various parts of the town, and the large amount of rubbish thrown into empty plots and on the streets interfere in water quality.

The areas around springs possess a strategic function in the movement of animals, providing ecological corridors [25] The Municipal Ecological Park of Mata da Bica, where several springs are situated, supplying the Preto River in the town of Formosa, is strategically placed for maintaining fauna since it lies between the Ecological Station of Aguas Emendadas (Planaltina, Federal District) and the Preservation Area of the Army Training Ground of Formosa. This makes a connection for birds that crosses the whole town, helping with the movement of fauna and dispersal of plant species. It is also worth highlighting that the Cerrado biome is on the list of the 25 world hotspots for biodiversity conservation [26]. Not only the alterations in the quality of the water but also in the structure of the vegetation can provoke significant impacts in the population dynamics of this group, mainly causing alterations in population and the disappearance of species.

### 3.2. Statistical Analysis

Initially, based on descriptive statistics, it was possible to observe the mean, maximum and minimum values and the standard deviation of the analyzed parameters among all those collected. These are shown in Table 4.

**Table 4:** Descriptive statistics attributed to physical, chemical and biological parameters.

Variables	Mean	Standard Deviation	Minimum	Maximum
**Water Temperature**	22.35	2.66	18.08	27.40
**pH**	7.15	0.64	5.81	7.85
**Conductivity**	134.6	37.90	35.00	168.00
**TDS**	67.32	18.92	18.00	84.00
**DO**	4.73	1.38	2.29	7.42
**Hardness**	44.32	20.58	9.00	81.00
**Turbidity**	2.52	1.61	0.02	5.29
**Coliforms**	2197.81	457.51	920.80	2419.60
***E.coli***	772.28	880.66	18.70	2419.60
**Sodium**	10.17	4.71	1.89	16.21
**Potassium**	1.31	0.28	0.34	2.33
**Calcium**	10.63	3.50	4.01	15.98
**Magnesium**	2.32	0.33	0.40	7.10
**Fluoride**	0.06	0.01	0.02	0.11
**Chloride**	4.63	2.53	0.66	8.72
**Nitrite**	0.03	0.06	0.00	0.24
**Nitrate**	3.33	5.58	0.00	13.85
**Sulfate**	0.82	0.85	0.30	3.80

With the exception of the nitrite variable, which had values detected only at point 2, the others were submitted to the normality test. From this, it could be seen that only the data on the variables total coliforms (*p* = 0.0009), magnesium (*p* = 0.02), nitrate (*p* = 0.02) and sulfate (*p* = 0.04) did not demonstrate normal distribution. Thus, these variables, including nitrite, were not submitted to analyses of variance (ANOVA) and clustering (Cluster).

Considering the variables with parametric data, *i.e*., those that presented normal distribution, it was possible to infer from the ANOVA test that only water temperature and turbidity presented significant differences between the months in which sampling was carried out. This demonstrated variation in function of seasonality, as observed in Table 5. The other variables did not demonstrate significant differences in their means at different sampling periods.

**Table 5:** Analysis of variance of means for variables between sampling periods (May, June, August and October).

Summary Anova		
Variables	F Value	*p* Value
***Water Temperature***	4.116	0.0275 *
***pH***	1.238	0.333
***Conductivity***	1.779	0.197
***TDS***	1.766	0.2
***DO***	0.52	0.675
***Hardness***	1.075	0.391
***Turbidity***	4.129	0.0272 *
***E.coli***	0.007	0.999
***Sodium***	0.084	0.968
***Potassium***	0.374	0.773
***Calcium***	3.234	0.0547
***Fluoride***	1.071	0.393
***Chloride***	0.287	0.834

Notes: (*) Value of p lower than 0.05, indicating significant differences based on the degree of freedom adopted (α = 5%).

As regards the collection locations, it was also through Analysis of Variance that the lack of significant variation could be observed in the variables water temperature, turbidity and F. Thus, specific environmental characteristics at each point may have influenced the variation in mean values for other variables, such as pH, conductivity, DO and TDS, at least at one of the five points (Table 6).

**Table 6:** Analysis of variance of means for variables between the sampling points (Points 1, 2, 3, 4 and 5).

Summary Anova		
Variables	F Value	*p* Value
***Water Temperature***	2.353	0.108
***pH***	16.39	5.37E-05 *
***Conductivity***	6.831	0.00345 *
***TDS***	6.841	0.00342 *
***DO***	13.41	0.000152 *
***Hardness***	38	4.69E-07 *
***Turbidity***	0.489	0.744
***E.coli***	74.46	7.91E-09 *
***Sodium***	90.64	2.33E-09 *
***Potassium***	46.31	1.44E-07 *
***Calcium***	6.263	0.00491 *
***Fluoride***	2.214	0.124
***Chloride***	37.32	5.22E-07 *

Notes: (*) Value of p lower than 0.05, indicating significant differences based on the degree of freedom adopted (α = 5%).

**Figure 6:**
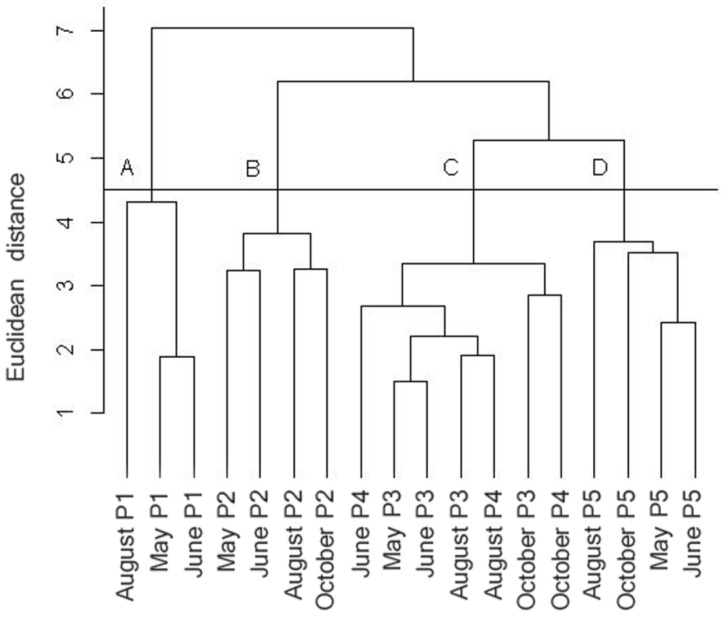
Dendrogram after Cluster analysis from the collected data.

The data that were standardized by means of the “standardize” function and transformed into a distance matrix by means of a Euclidean similarity index were submitted to Cluster analysis and resulted in a dendrogram. This, in turn, was analyzed with abline at point 4.5, demonstrating the formation of four groups (A, B, C and D) containing the points in each collection month. M1, M2, M3 and M5 are the points 1, 2, 3 and 5 collected in May; J1, J2, J3, J4 and J5 are the points 1, 2, 3, 4 and 5 collected in June; A1, A2, A3, A4 and A5 = collection of the five points in August; and O2, O3, O4 and O5 are the collection in October at points 2, 3, 4 and 5 (Figure 6).

As observed in Figure 6, group A includes point 1 in all its sampling periods, as does group B, formed by the grouping of point 2 in the four monitoring periods. Group C includes points 3 and 4 throughout the collection months. Group D, in turn, is formed by point 5.

It can thus be inferred that the grouping of collection points is due to the standard of the variables analyzed at each locality, with a significant value of cophenetic correlation, and Pearson’s r equal to 0.85. As indicated by Bocard *et al.* [14] a coefficient of cophenetic correlation over 0.7 indicates a good correlation between the variables that characterize the standard grouping obtained.

Analyzing the influence of temporal variation on the configuration of the groups, subgroups can be identified among groups A, B, C and D, conditioned by the factor of collection time. There is a greater similarity between the months of May and June in Group A. The second group, referring to Point 2, presents two more similar periods: a grouping of the months of May and June, and another one with the months of August and October. Group C, in turn, is subdivided into two groups, the first including Points 3 and 4 in the months of May, June and August, and the second with both points in the month of October. Finally, in group D, as with the previous groups, there is a shorter Euclidean distance between the months of May and June, followed by October and August.

As regards collection points, the analysis of variance made it possible to observe greater significant differences between the variables except for water temperature, turbidity and F^-^, which did not demonstrate a significant difference. The result of ANOVA carried out between the collection points corroborated the result of Cluster analysis, and this in turn allowed us to identify a standard grouping of points in function of the variables.

Thus, the characterization of Point 1 (Group A—Figure 6), water body spring, shows that it is most different from the rest of the points, possibly because of a configuration that is more favorable to maintaining water quality, such as the existence of riparian forest. Point 2, localized in a channeled region and in contact with untreated effluents (Group B), is also in contrast to the other points, in that it presents compromised water quality that is more evident than at the other points.

Points 3 and 4, grouped together, may present more similar characteristics because of their spatial proximity, which provides similar environmental conditions. Lastly, it was possible to observe grouping at point 5 in the four sampling periods into a single group (Group D), allowing an inference about the influence of the lentic environment upstream from the sampling location.

It can thus be observed that spatial distribution was more significant for the formation of groups than the collection period. The points therefore present greater differentiation among themselves than in function of the temporal variation recorded throughout the study.

Based on the obtained results, it is confirmed that urban occupation near the headwaters of the Preto River watercourse in the town of Formosa (P2) has led to alterations in the natural characteristics of the water.

Josefa Gomes creek underwent several impacts, including elimination of gallery forests, buildings in riparian areas, soil sealing, concentrated release of storm water, untreated wastewater discharge from clandestine inputs, release of solid waste and waste of different nature, erosion formed by leaching, stream bed obstruction by dumping of waste and soil deposit generated by silting processes.

The input of waste from the small watershed (storm drainage) located in the urban area and flows into the Feia Lagoon contribute to the silting process. Whereas the urban area of Formosa city is almost entirely located in the study area, water bodies in this catchment zone are subject to the effects of different sources of pollution and intrinsic forms of degradation of a city that had a population growth and expansion of occupied areas without planning.

This scenario reflects the poor planning in use and occupation, and is mainly due to the insufficient infrastructure and environmental sanitation and urban drainage in Formosa city. Throughout the river basin under study there are environmental impacts such as degradation of the surface water quality, aquatic weeds proliferation, losses in aquatic life, landscape degradation and smelly water. Several countries have suffered problems with rapid urbanization of natural areas. Water is a natural resource essential to all living beings, and preserving their quality is to ensure life. This is just one of the great challenges in various cities throughout the world today; to develop sustainably, while preserving the regions of springs and ponds that run through urban centers [27].

Although in this study the number of collections was small throughout the year, it was possible to observe natural characteristics of the river, based on the P1 data, and the impact of urbanization in these values when looking at the P2 data. This diagnosis is essential to know and seek to minimize the consequences of this impact on the natural resource and subsequently on human health.

## 4. Conclusions 

Point P2 is located soon after Beira Rio Creek (which is channeled), and this stretch of the water does not have contact with riparian soil and forest. It probably also receives domestic sewage, and for these reasons presented high values of various parameters such as F^−^, SO_4_^2−^, NO_3_^−^ and Na^+^ compared to the other points. In addition, the large number of aquatic plants after P2 reinforces the hypothesis that water coming from the creek is eutrophicated.

The presence of aquatic plants after P2 throughout the headwaters of the Preto River may be responsible for consumption of nutrients such as NO_3_^-^ and K^+^, as well as for filtering some of the contaminants that come from upstream. If the source of nutrients is not interrupted, there may be an increase in the number of plants, and macrophytes may occupy the whole water body.

The headwaters of the Preto River in Formosa, Goiás State, Brazil have been undergoing negative environmental impacts due to urban expansion that is taking place in an uncontrolled manner along riverbanks and watercourses. The data presented on water quality in the headwaters of the Preto River at Formosa constitute a warning to society and to public policymakers in this town, highlighting the current situation and the need to take measures to deal with the contamination and pollution of this water body.

To reverse the current degradation framework is required action planning for sustainable use of natural resources of the watershed of Feia Lagoon.
